# Altered attention network dynamics in resting-state fMRI of individuals with high smartphone addiction scores

**DOI:** 10.3389/fnins.2026.1948718

**Published:** 2026-10-14

**Authors:** Harrison Watters, Aida Abdul Rashid, Subapriya Suppiah, Theodore J. LaGrow, Jaroslav Hlinka, Shella Keilholz

**Affiliations:** 1 Institute of Computer Science of the Czech Academy of Sciences, Prague, Czechia; 2 Department of Psychological Science, Weber State University, Ogden, UT, United States; 3 Center for Diagnostic Nuclear Imaging, Universiti Putra Malaysia, Serdang, Malaysia; 4 Wallace H. Coulter Department of Biomedical Engineering, Georgia Tech and Emory University, Atlanta, GA, United States

**Keywords:** attention networks, attractor states, default mode network, dynamics, quasi-periodic patterns, resting state fMRI, smartphone addiction

## Abstract

**Introduction:**

Altered attention network activity has been consistently reported in recent neuroimaging literature of smartphone addiction and problematic smartphone use. However, many functional imaging studies have relied on traditional static functional connectivity analyses, which cannot capture time-varying or recurrent dynamics of attention networks.

**Methods:**

Given the importance of temporal and spatial dynamics for attentional processing, we applied two complementary dynamic functional connectivity approaches: functional connectivity-based attractor networks (fcANN), which characterize transitions between discrete attractor states related to internal-external context and action-perception dynamics, and cPCA-based quasi-periodic pattern (QPP) analysis, which captures phase relationships between cortical networks including the default mode network, dorsal attention network, and frontoparietal control network.

**Results:**

In a resting-state fMRI dataset comparing participants with High and Low SAS-M scores (SAS-M: Smartphone addiction scale-Malay version questionnaire), we observed context-dependent differences in DMN-attention-network relationships across attractor states. The most consistent effects emerged during transitions between internal and external attractor states, where subjects with high smartphone addiction (SPA) scores showed greater DMN dominance relative to attention-related networks. For DMN-FPN, these differences correlated with subject SAS-M scores.

**Discussion:**

Overall, these findings provide preliminary evidence that smartphone addiction may be associated with altered large-scale attentional dynamics at rest, warranting further investigation in larger and more diverse samples.

## 1 Introduction

People commonly use smartphones for 3–10 h per day in high income economies (McCoy, 2020; Brodersen et al., 2022; Marciano and Camerini, 2022; Coyne et al., 2023; Nurbaiti, 2023; Tomczyk and Selmanagic Lizde, 2023). Assuming 8 h of sleep per night (optimistic), this means many humans now spend more than half of their waking life engaged with smartphones; and about half of that on social media (Coyne et al., 2023; Tomczyk and Selmanagic Lizde, 2023). The shift toward obligate digital attention accompanies significant post-industrial changes in work-related physical activity, with many office and screen-oriented workers reaching sedentary screen time upwards of 15 h per day (Levine, 2015; Jain et al., 2023).

These abrupt digital changes to human lifestyle are associated with numerous adverse health effects on sleep, metabolism, vision, and overall physical health (Nakshine et al., 2022), as well as elevated risk of psychiatric disorders such as depression, suicidal ideation, anxiety, narcissism, and attention deficit hyperactivity disorder (Vahedi and Saiphoo, 2018; Giordano et al., 2019; Kim et al., 2019; Ksinan et al., 2021; Brodersen et al., 2022; Nakshine et al., 2022). The behavioral correlates of digital overload, specifically smartphone addiction, are generally bleak. These behavioral effects raise an important question regarding their neural correlates. In terms of historical context, human brains evolved over several million years of foraging and hunting in small, tightly knit communities - markedly different from the modern information landscape (Porzio, 2020; Burr, 2025). Naturally, there is significant interest in the *cognitive* cost of the devices, such as smartphones, which are shaping our modern social environment (Korte, 2020).

Neurofunctional changes associated with internet and smartphone addiction (SPA) are now widely reported (Lin et al., 2022; Anbumalar and Binu Sahayam, 2024). Predictably, association networks involved in cognition, executive function, and goal-directed external attention are among the most consistently implicated (Wang et al., 2019; Lee et al., 2021; Han and Kim, 2022; Kwon et al., 2022; Schmitgen et al., 2022; Áfra et al., 2024). However, cortical networks are shaped by mechanisms of plasticity throughout the lifespan, tuning connectivity to reflect environmental demands (Soares et al., 2013; Posner et al., 2014; Swingler et al., 2015; Power and Schlaggar, 2017; Klein et al., 2024). Lifelong neural plasticity complicates interpretation of whether smartphone-related neurofunctional changes should be viewed as maladaptive, adaptive, or context-dependent. While there is evidence that smartphone use can impair cognition and working memory (Ward et al., 2017; Stone, 2020; Akkurt and Aksoy, 2025), others argue that media-multitasking can improve attention (Kobayashi et al., 2020). Perhaps our brains are simply doing what they do best: adapting. In experienced non-smartphone platform videogamers for example, task-related activation in attention networks is altered, but the changes are associated with *improved* reaction times in visuospatial tasks (Jordan and Dhamala, 2023), and even short-duration gaming is linked to improved visual attention and executive function in elderly subjects (McCord et al., 2020). However, such improvements in performance may plateau, as video game experience improves multitasking only up to intermediate levels of difficulty and does not appear to protect against the effects of heavy media multitasking (Cardoso-Leite et al., 2016). In other words, plasticity can only compensate for so much multitasking.

Certain types of video game experience involve prolonged focus and goal-directed problem solving, however, it is unclear whether extensive smartphone use, particularly with regard to social media, has any such adaptive benefit (León Méndez et al., 2024). Importantly, however, SPA is not synonymous with problematic social media use and may reflect heterogeneous patterns of smartphone engagement, including social media, gaming, and other applications. At the broader level of SPA, emerging evidence suggests maladaptive changes resembling general features of addiction, including volume changes in the anterior cingulate cortex (ACC), a key region in motivation and cognitive control, as well as cue-related dopaminergic activity (i.e., craving) in the nucleus accumbens (NAcc), a primary rewards center of the brain (Parvizi et al., 2013; Schmitgen et al., 2020; Abdul Rashid et al., 2021; Henemann et al., 2023), and changes in precuneus activity related to impaired attention (Nasser et al., 2020). Excessive screen time on mobile digital devices has also been associated with reductions in cortical gray matter volume, with some authors suggesting possible implications for neurodegenerative risk later in life (Manwell et al., 2022).

While task- and resting-state functional MRI (rs-fMRI) studies have identified SPA-related alterations in attention and executive-control networks, most of the aforementioned work has relied on traditional static functional connectivity (FC) measures, in which relationships between regions of interest (ROIs) are averaged across an entire scan. Such approaches provide useful estimates of overall network organization, but do not directly characterize time-varying fluctuations or temporal phase relationships between networks (Keilholz et al., 2017; Bolt et al., 2022; Meyer-Baese et al., 2022). Because attentional processes fluctuate over time and across cognitive contexts, dynamic FC approaches may provide complementary information regarding large-scale network coordination in SPA. Indeed, there is evidence that phase information (temporal offset between network activations) might be more related to cognitive state than average activation or functional connectivity (Song et al., 2026).

To capture brain dynamics in a dataset of resting-state SPA subjects, we combined two approaches: functional connectivity-based attractor networks (fcANN) and quasi-periodic pattern (QPP) analysis. The attractor network approach, implemented by Englert et al. (2025), captures dynamic brain states as free-energy minimizing attractor basins analogous to Hopfield (1982) Networks. The fcANN approach was found to be robust across datasets, sites, and acquisition parameters to reliably characterize four dynamic brain states in resting-state scans corresponding to two interpretable axes: internal vs. external context, and action vs. perception states (Englert et al., 2025). This approach offers the advantage of capturing task-like brain states in resting-state time series. Using their approach on a resting-state dataset of autism spectrum disorder (ASD), for example, they were able to detect alterations to sensory and default mode network (DMN) areas consistent with perceptual changes in ASD.

Applying QPP analysis offers complementary insight, whereby cycles of anticorrelated activity between networks can be detected across imaging modalities (Majeed et al., 2011; Thompson et al., 2014; Xu et al., 2025). Although QPP analysis can capture anticorrelated activity between the DMN and externally oriented attention networks, such anticorrelation should not be interpreted as a strict functional dichotomy or “task-positive” vs. “task-negative,” as DMN activity can also contribute to goal-directed cognition (Spreng, 2012). QPPs occur on various timescales, but are typically described as infraslow (∼0.05–0.1 Hz) cycles, meaning swells of network activity, lasting ∼20 s in group-averaged time series (Yousefi et al., 2018; Maltbie et al., 2022). While QPPs are robust across task and rest states, QPP network fluctuations appear flexible based on task demand (Watters et al., 2025). QPPs are largely driven by activity in the DMN, which is implicated in mind wandering, cognition, and memory recall (Raichle, 2015). DMN activity is typically antiphase with the dorsal attention network (DAN), which flexibly interacts with other networks such as the ventral attention network (VAN) and visual networks (VIS) to mediate externally oriented or goal-directed attention (Corbetta and Shulman, 2002; Vossel et al., 2014; Rajan et al., 2021; Zhao et al., 2022). The DAN in particular is a natural target of interest in SPA given its role in distractor suppression (Lanssens et al., 2020). Lastly, the frontoparietal network (FPN), sometimes called the executive control or control network (Witt et al., 2021) is associated with executive function and information selection (Scolari et al., 2015; Marek and Dosenbach, 2018), and has demonstrated reduced activity during distractor tasks among smartphone addicted subjects, with corresponding failures of executive control (Han and Kim, 2022). The balance of activity and phase offset between cortical networks such as the DMN, DAN, and FPN during QPP events appears to be important to attention and task performance (Seeburger et al., 2024, 2026). There is additional evidence that QPP dynamics are disrupted in disorders of sleep and attention (Abbas et al., 2019; Daley et al., 2025). Applying QPP analysis in SPA is thus a natural extension of the method.

Each of these dynamic approaches provides distinct but related information about large-scale brain organization. The attractor-network framework characterizes recurring brain states and transitions between attentional contexts, including internal versus external orientation and action versus perception-related states. QPP analysis, in contrast, characterizes recurring spatiotemporal patterns and temporal phase relationships between cortical networks, including interactions involving the default mode and attention systems. Applied together, these approaches permit examination of how large-scale network phase relationships vary across brain states and transitions during resting-state activity. Prior work has shown that dynamic relationships between the DMN and attention networks, including the DAN and FPN, vary systematically across internally and externally oriented attentional contexts and are associated with individual differences in attentional control (Watters et al., 2025; Seeburger et al., 2026). We therefore hypothesized that individuals with high SAS-M scores would exhibit reduced anticorrelation between the DMN and the DAN and/or FPN during QPP-like dynamics. We further hypothesized that these differences would be apparent surrounding transitions between internally and externally oriented attractor states, consistent with altered coordination between default-mode and attention networks during shifts in large-scale brain state.

This study presents an exploratory application of combined attractor-state and QPP analyses to characterize resting-state network dynamics in relation to high smartphone addiction scores (SAS-M). Using fcANN, we examined whether SPA was associated with altered occupancy and transition structure across attractor-defined attentional states, including internal versus external and action versus perception-related modes of network organization. Using QPP analysis, we further examined whether SPA was associated with altered phase relationships among large-scale resting-state networks, particularly involving the DMN, DAN, FPN, and related sensory-attentional systems. Finally, by relating QPP dynamics to attractor-defined states and transitions, we examined whether large-scale network phase relationships varied systematically across different modes of resting-state brain organization.

## 2 Materials and methods

### 2.1 Participants and dataset

Resting-state fMRI data included 45 participants organized by the original study team into High SAS-M (*n* = 21) and Low SAS-M (*n* = 24) groups. These supplied SAS-M group assignments were retained for the present study. Five participants were excluded during preprocessing quality control because of excessive head motion or gross spatial-registration failure (three High SAS-M and two Low SAS-M), yielding a final sample of 40 participants (High SAS-M: *n* = 18; Low SAS-M: *n* = 22). The resulting score distributions were consistent with the previously validated SAS-M screening threshold of >98 for identifying individuals at elevated risk of problematic smartphone use (Ching et al., 2015; Figure 1A). Note that the SAS-M is a screening instrument rather than a clinical diagnostic assessment, thus, the groups are referred to as High and Low SAS-M in the manuscript. Although this previously validated threshold for SAS-M score provides a principled basis for the supplied group classification, SAS-M scores represent a continuous self-report measure; accordingly, complementary continuous associations between SAS-M scores and imaging-derived measures were also examined.

**FIGURE 1:**
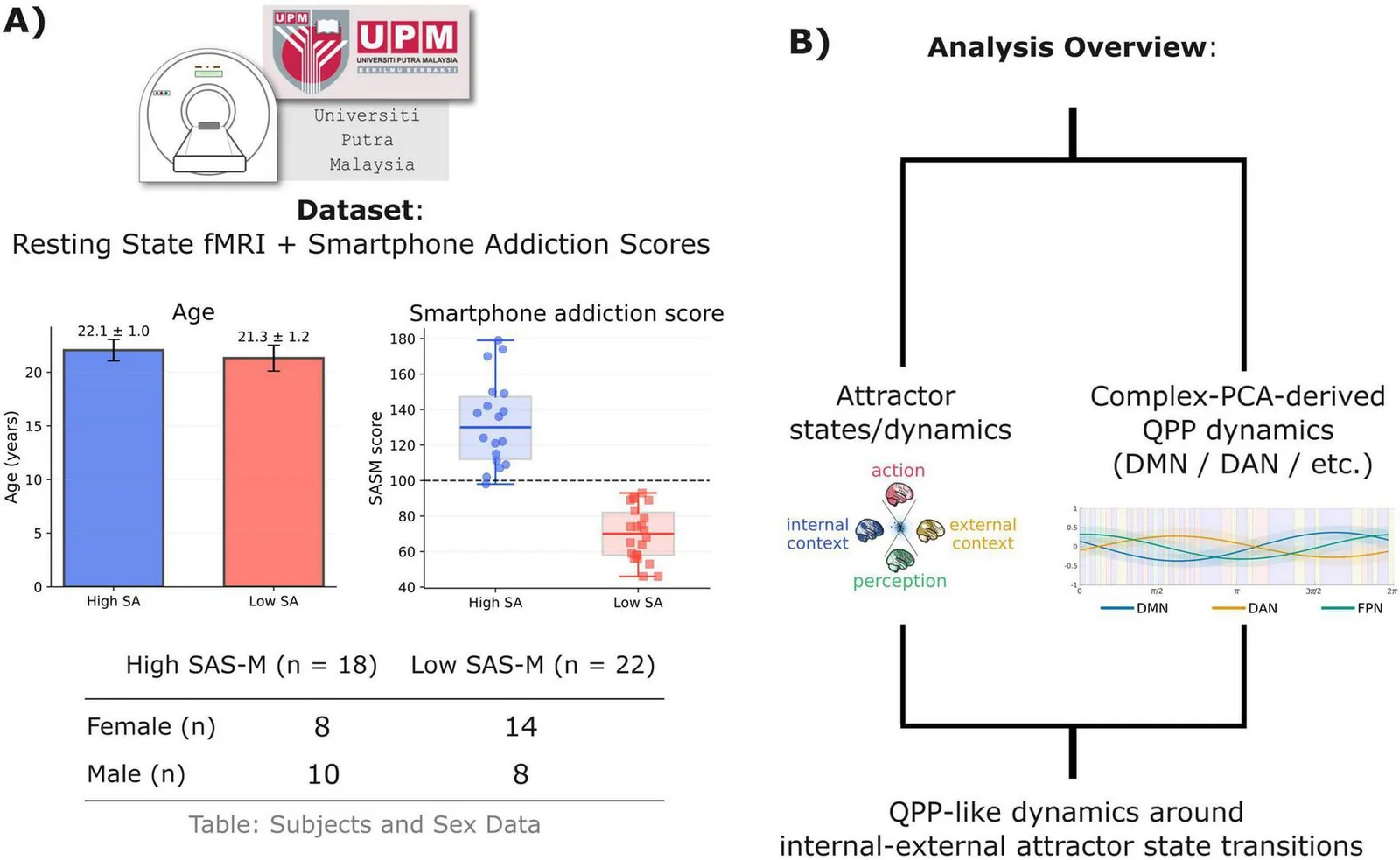
Study and Analysis Overview. **(A)** Dataset characteristics and High and Low SAS-M groups. **(B)** Overview of the two dynamic analysis approaches. Functional connectivity-based attractor networks (fcANN) characterize large-scale attractor states and their dynamics, whereas cPCA/QPP analysis identifies recurring spatiotemporal patterns and their network-level expression. QPP dynamics were subsequently examined in relation to and surrounding attractor-state transitions.

All participants were right-handed, had normal or corrected-to-normal vision, and reported no history of neurological or psychiatric disorders. The groups did not differ significantly in sex distribution (two-sided Fisher’s exact test, *p* = 0.339); sex and age counts are shown in Figure 1.

The original study was approved by the Ethics Committee of Research Involving Human Subjects of Universiti Putra Malaysia. All participants provided written informed consent. The present study constitutes a secondary analysis of previously acquired data.

### 2.2 MRI data acquisition

MRI data were acquired on a 3T scanner (PRISMA, Siemens, Erlangen, Germany).

Structural MRI scans were acquired with the following parameters: High-resolution T1-weighted magnetization-prepared rapid gradient echo (MPRAGE) sequence. Repetition time (TR) of 2,300 ms, echo time (TE) of 2.27 ms, and inversion time (TI) of 1,100 ms. Images were collected in an ascending sagittal orientation with 160 slices, a field of view (FOV) of 256 × 256 mm^2^, matrix size of 256 × 256, isotropic voxel resolution of 1 mm.

Resting-state functional images were obtained using a gradient-echo echo-planar imaging (EPI) sequence sensitive to blood-oxygen level dependent (BOLD) contrast. The phase encoding direction was anterior-to-posterior. Functional acquisition parameters included a repetition time (TR) of 3,000 ms and an echo time (TE) of 30 ms, with 256 TRs, giving a total scan length of ∼13 min per subject. Whole-brain coverage was achieved with 34 slices of 3 mm thickness, a field of view (FOV) of 220 × 220 mm^2^, and a voxel size of 2.3 × 2.3 × 3 mm. Whole-brain functional images were collected during resting-state with participants instructed to remain still with their eyes closed but not fall asleep, and not engage in any specific task.

### 2.3 Resting-state preprocessing

Functional MRI data were preprocessed using the CONN functional connectivity toolbox (Whitfield-Gabrieli and Nieto-Castanon, 2012) implemented in MATLAB, which relies on SPM-based routines. Preprocessing followed a standard pipeline broadly comparable to that used in the original study, with minor differences in implementation.

Preprocessing steps included: Slice-timing correction to account for temporal offsets between slices, realignment (motion correction) using rigid-body transformation, coregistration of functional and anatomical images, segmentation of anatomical images into gray matter, white matter, and cerebrospinal fluid (CSF), normalization to the MNI-152 standard space, and spatial smoothing. Denoising (in CONN) included: regression of motion parameters, regression of physiological noise using principal components from white matter and CSF signals (CompCor approach), and linear detrending and temporal filtering (band-pass, 0.008–0.09 Hz).

Global signal regression (GSR) was not performed; thus, variance associated with the whole-brain global signal was retained following the preprocessing steps described above. The decision to retain the global signal was also motivated by prior cPCA work demonstrating that globally synchronous activity and QPP-like spatiotemporal dynamics can emerge as separable components, with the canonical QPP typically represented by a dominant non-global component (Bolt et al., 2022). Thus, cPCA permits characterization of QPP dynamics without requiring explicit removal of the global signal during preprocessing.

These preprocessing steps are broadly equivalent to those used in the original study (which employed SPM-based preprocessing), ensuring comparability of the resulting time series.

All time series were parcellated to BASC-122 atlas space before analysis (Bellec et al., 2010). This 122 functional parcel atlas was selected to match the process used by Englert et al. (2025) for their Attractor Network approach, which was optimized on the same atlas.

### 2.4 Comparisons and correction

Group comparisons were conducted using two-sided Wilcoxon rank-sum tests and subject-level permutation tests, as specified for each analysis. Multiple comparisons were controlled using the Benjamini-Hochberg false-discovery-rate procedure where stated, with q < 0.05 considered significant. Effect sizes for between-group comparisons were quantified using Cohen’s *d* and, where reported, Cliff’s delta. Rank-sum and permutation-derived *p*-values are identified separately. Uncorrected results are presented only for exploratory interpretation and should be considered hypothesis-generating.

### 2.5 Attractor state detection

Attractor states were identified using the *connattractor* framework (Englert et al., 2025, PNI Lab), which implements a Hopfield neural network-based model of whole-brain functional connectivity dynamics. This approach uses a pretrained embedding derived from a large multi-site dataset, in which canonical brain states are defined as stable attractors in an energy landscape learned from functional connectivity patterns. This embedding was derived from an independent dataset [by Englert et al. (2025)] and was not trained on the present sample. Full methodological details and validation of this framework are described in the original study (Englert et al., 2025), which demonstrated that these attractor states generalize across datasets, acquisition protocols, and temporal resolutions.

For the present study, preprocessed resting-state fMRI time series (BASC-122 parcellation) were projected into this pretrained attractor embedding using the default template provided by the connattractor package. At each time point (TR), the whole-brain activity pattern was evaluated within the Hopfield network to compute its corresponding energy and to determine its proximity to canonical attractor states. Each time sample was then assigned to the nearest attractor using connattractor functions *get_attractors_per_timesample* and *label_att_states*, resulting in a discrete time series of attractor labels for each participant. The original template defines multiple attractor states; for interpretability and consistency with prior work by Englert et al. (2025), these were remapped to four canonical states reflecting two major cognitive axes (internal context, action, external context, and perception). For a sense of the cognitive axes, see Figure 2A.

**FIGURE 2:**
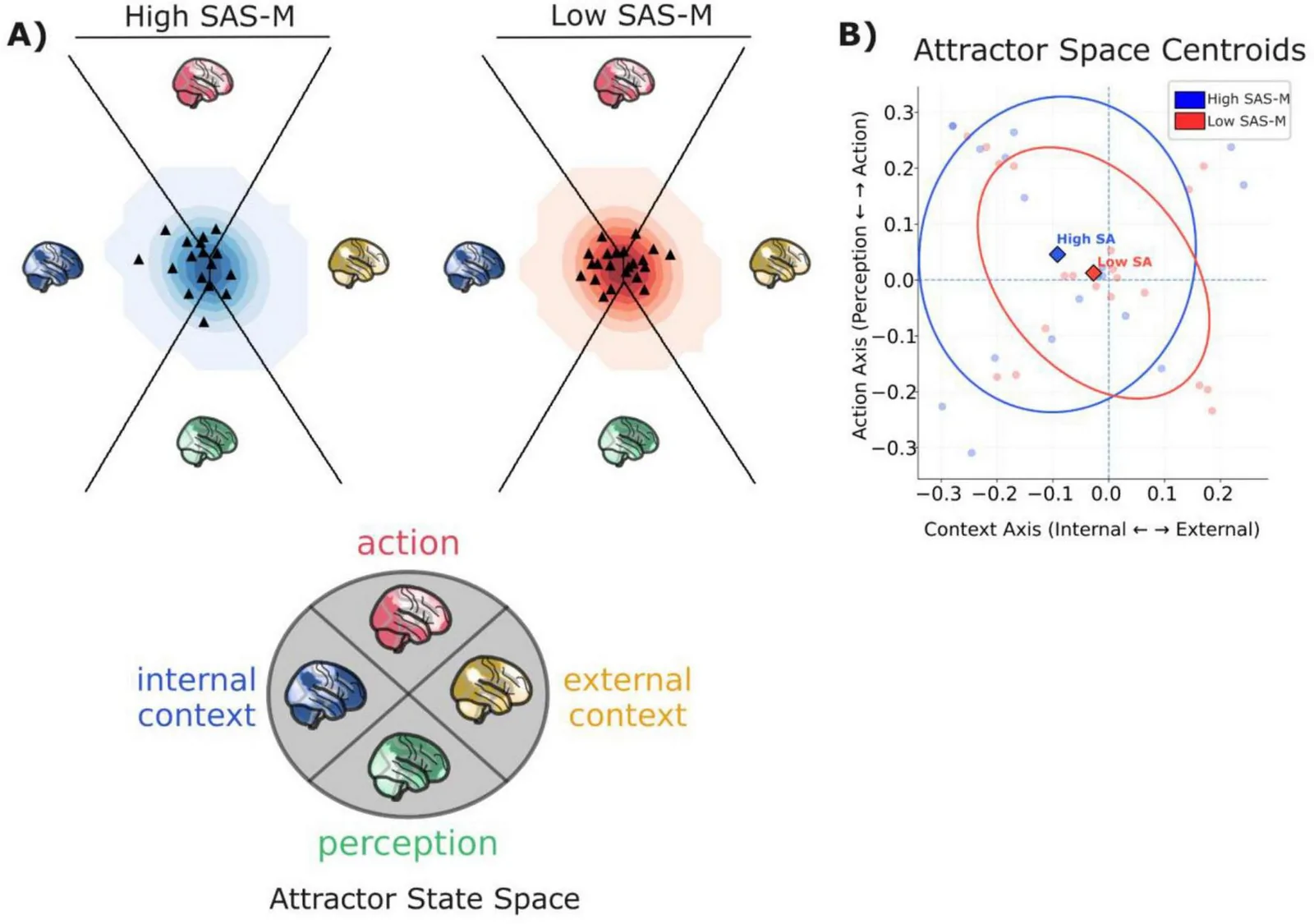
Attractor state-space density plots between groups. Whole-brain activity time series were projected into a shared two-dimensional space defined by the fcANN embedding. The horizontal axis represents the internal-external context gradient, and the vertical axis represents the perception-action gradient. **(A)** State-space density maps show the distribution of time points for the High and Low SAS-M groups. Darker regions indicate greater time-point density. Black triangles indicate individual participants’ mean positions. **(B)** Subject-level mean positions are displayed at an expanded scale to show variation near the origin. Diamonds indicate group centroids, ellipses represent approximately 1.5-standard-deviation covariance contours, and dashed lines mark the origin. The High SAS-M group showed greater descriptive dispersion and a small shift toward the internal-context and action directions; these visual differences were descriptive and were not subjected to formal inferential testing.

### 2.6 Attractor state metrics

Following attractor state assignment, summary metrics were derived at both the time point and subject levels (Figure 3). For each participant, the fraction of time spent in each attractor state (state occupancy) was computed, along with entropy and switch rate. These state labels and associated metrics were subsequently used to examine relationships between large-scale brain network dynamics, quasi-periodic patterns (QPPs), and behavioral measures.

**FIGURE 3:**
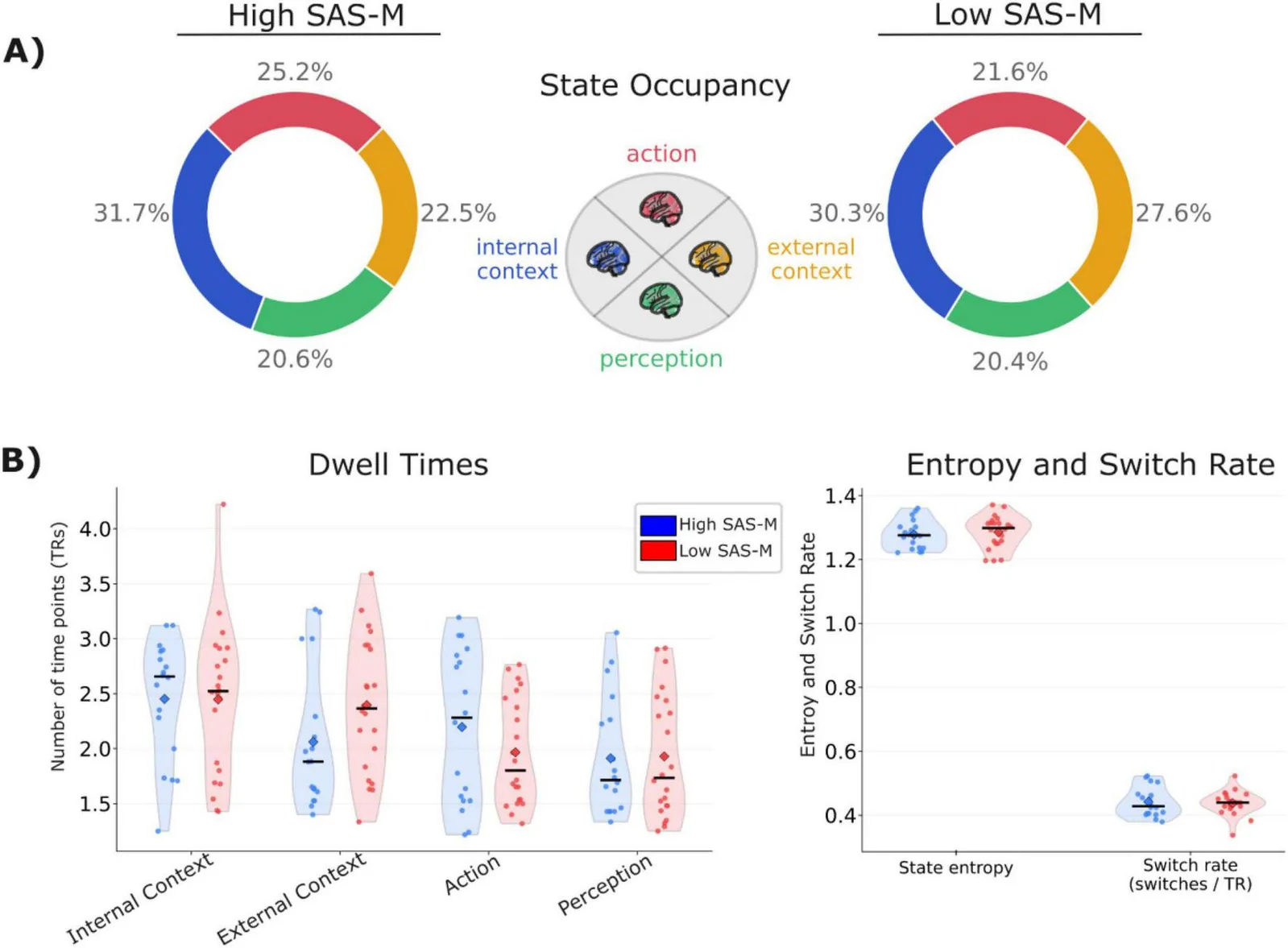
Attractor state dynamics. **(A)** Fraction of time (TRs/time points) spent in each attractor basin; internal - external, action - perception by group, High SAS-M, high smartphone addiction scores, Low SAS-M, low smartphone addiction scores. **(B)** Additional attractor dynamic metrics including: mean dwell time within (from left to right) internal context, external context, action, and perception states, and in bottom right the entropy and switch rate (changes of state per TR). The four-state occupancy profiles **(A)** did not differ significantly between groups in the group label omnibus permutation test (*p* = 0.454; 10,000 permutations). Subject-level group comparisons of dwell time, entropy, switch rate, and axis metrics were assessed using two-sided Wilcoxon rank-sum tests.

For attractor-state summary metrics (Figure 3), group differences were assessed at the subject level using two-sided Wilcoxon rank-sum tests comparing High SAS-M (*n* = 18) and Low SAS-M (*n* = 22) participants for state fractions, context/action-axis summary scores, mean dwell times, state entropy, and switch rate. For descriptive interpretation, effect sizes were quantified as Cohen’s *d* and Cliff’s delta. Levene’s test (center = median) was additionally computed to assess heterogeneity of variance between groups for each metric. These analyses were performed on one value per subject for each metric.

To assess overall state occupancy in the donut plots (Figure 3), we performed an omnibus permutation test on the subject-wise occupancy vectors, using 10,000 random label permutations to obtain the null distribution.

### 2.7 Exploratory attractor state-specific regional activity comparisons

State-specific regional activity was quantified by averaging parcel-wise BOLD time series within each subject and attractor state (Figure 4), yielding one mean value per BASC-122 parcel, state, and subject. These analyses were conducted to exploratorily characterize potential state-specific group differences and were not intended as an omnibus test of Group × State × Network effects.

**FIGURE 4:**
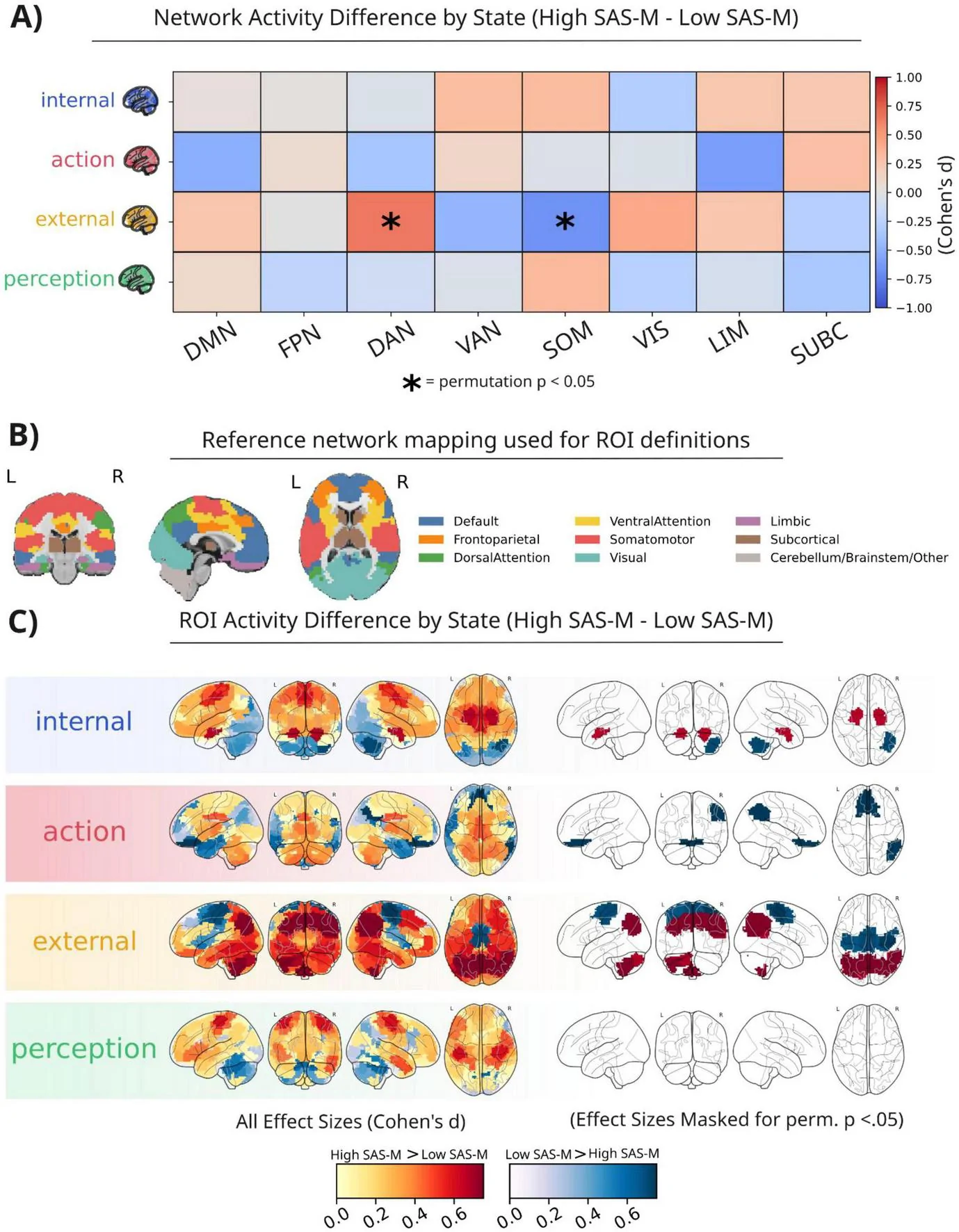
State-specific activity changes (High SAS-M and Low SAS-M) by region (networks and parcels). State-specific regional activity was averaged within subject, state, and parcel prior to group comparison. Between-group differences (High SAS-M vs. Low SAS-M) were assessed using subject-level permutation tests (10,000 permutations), with Cohen’s *d* shown as effect size (High SAS-M minus Low SAS-M). Multiple comparisons were controlled within each state using BH-FDR. Maps are shown unthresholded and thresholded at nominal (*p* < 0.05) levels; no effects survived FDR correction. **(A)** Network-level differences in mean BOLD activity across attractor states (internal context, action, external context, perception), shown as Cohen’s *d* effect sizes (High SAS-M minus Low SAS-M) for major attention networks (e.g., DMN, DAN, FPN, VIS). *Indicates an uncorrected permutation *p* < 0.05; no comparisons survived BH-FDR correction. **(B)** Approximate mapping between BASC-122 parcels and canonical large-scale networks used for network-level aggregation. **(C)** Parcel-wise activity differences (High SAS-M−Low SAS-M) visualized across the cortical surface for each attractor state. Warm colors indicate greater activity in the High SAS-M group, while cool colors indicate greater activity in the Low SAS-M group. Right panels show thresholded maps (permutation *p* < 0.05, uncorrected). Although several regional effects reached uncorrected significance, none survived correction for multiple comparisons (BH-FDR), and results should be interpreted as exploratory.

Group differences (High SAS-M vs. Low SAS-M) were assessed independently within each attractor state using subject-level permutation tests (10,000 permutations of group labels), with the difference in group means as the test statistic and Cohen’s *d* (High minus Low SAS-M) as the effect-size measure. Analyses were performed at both the parcel level (122 BASC parcels) and network level, with parcels grouped into eight large-scale systems (DMN, FPN, DAN, VAN, VIS, SOM, LIM, SUBC). Benjamini-Hochberg FDR correction was applied separately across parcels or networks within each state.

For visualization, parcel-wise effect-size maps are shown both unthresholded and at nominal significance (permutation *p* < 0.05). Because no parcel- or network-level comparisons survived FDR correction, nominally significant effects are presented for exploratory purposes only. Subjects were treated as independent observations, and estimates were not weighted by the number of TRs contributing to each state.

### 2.8 Quasi-Periodic pattern detection

Complex principal component analysis (cPCA) was used to identify QPP-like components from rs-fMRI data, following the framework described by Bolt et al. (2022) and implemented in the publicly available *complex_pca* toolbox. In brief, the approach transforms preprocessed ROI time series into complex-valued analytic signals using the Hilbert transform, after which principal component decomposition is applied to the complex data matrix. This yields component time courses and complex spatial weights that jointly capture both the magnitude and relative phase structure of large-scale BOLD dynamics. Unlike conventional PCA, cPCA is specifically designed to represent time-lagged relationships among signals, allowing the recovery of propagating or anti-correlated spatiotemporal patterns rather than purely zero-lag covariance structure. Bolt et al. (2022) showed that this approach provides a compact description of resting-state fMRI dynamics and that one of the dominant components recovers QPP-like modes of anti-correlation between DMN and DAN areas.

In the present study, cPCA was first applied at the group level for exploratory visualization and comparison by concatenating scans within each group to generate average QPPs (Supplementary Figure 3). All subsequent primary QPP analyses were conducted at the subject level, with the principal network-resolved results presented in Figure 5 and supporting analyses provided in the Supplementary material.

**FIGURE 5:**
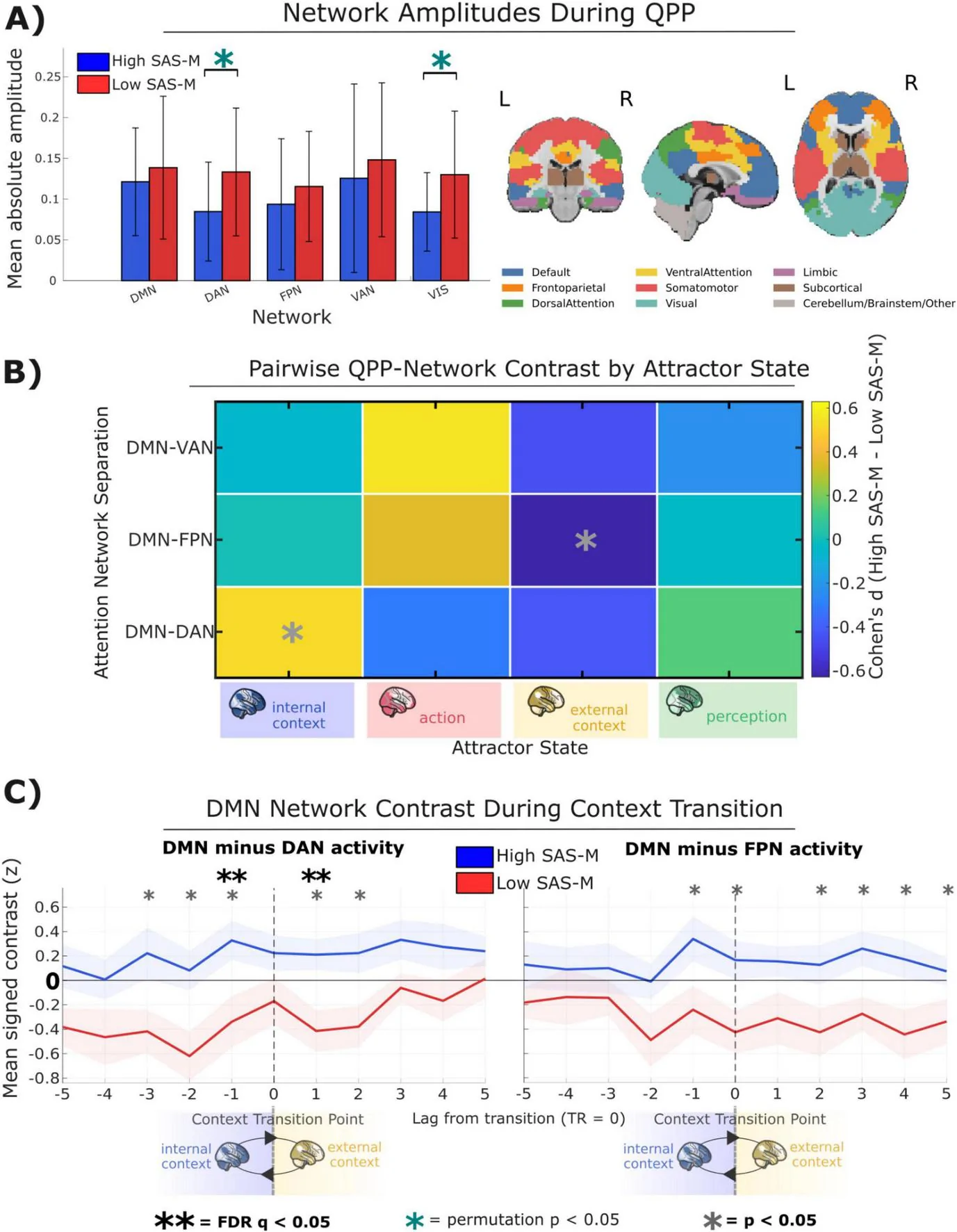
Attention network activity during QPPs, attractor states, and context transitions. Symbols indicate statistical significance as follows: gray *nominal uncorrected *p* < 0.05; teal *permutation *p* < 0.05; black **FDR-corrected q < 0.05. **(A)** Mean absolute QPP amplitude in five networks (DMN, DAN, FPN, VAN, VIS) by SAS-M group; error bars indicate SEM. DAN, and VIS amplitudes were lower in the High SAS-M group at nominal permutation-test thresholds (*p* = 0.032 and 0.037, respectively; 10,000 permutations), but neither difference survived FDR correction across the five networks. **(B)** Pairwise network separation (difference in QPP signal magnitude between networks) as a function of attractor state. Heatmaps display Cohen’s *d* effect sizes (High SAS-M−Low SAS-M) for DMN-centered contrasts (e.g., DMN-DAN, DMN-FPN, DMN-VAN) across the four attractor states (internal context, action, external context, perception). Asterisks indicate nominal significance (*p* < 0.05, uncorrected). The High SAS-M group showed nominally increased DMN-DAN separation (*p* = 0.033) and decreased DMN-FPN separation (*p* = 0.04) compared to the Low SAS-M group. **(C)** Transition-triggered analysis of network dynamics during shifts along the internal-external context axis. Internal-external analyses included 34 participants (18 High SAS-M, 16 Low SAS-M). Participants were included only when a complete transition window for that context was available. Plots show mean signed differences in activity between DMN and attention networks (DMN-DAN, DMN-FPN) as a function of time relative to transition onset (lag in TRs). Shaded regions indicate standard error. Significant timepoints (*p* < 0.05 and q < 0.05 after FDR correction across lags) are marked. Results indicate increased DMN dominance relative to attention networks in the High SAS-M group during transitions between internal and external states. Nominal uncorrected effects are shown for exploratory purposes and are visually distinguished from permutation and FDR-corrected results.

### 2.9 Subject-wise identification of QPP-like components

Unlike prior cPCA studies performed on concatenated group-level resting-state data (Bolt et al., 2022), the present study estimated QPP-like modes of activity independently within each subject in order to preserve inter-subject variability and permit subject-level statistical inference. While the canonical DMN-TPN pattern is consistently recovered as the dominant non-global component in concatenated datasets (i.e., component 2), the ordering and relative prominence of components varies at the subject level. Accordingly, subject-wise analyses required an additional component selection procedure to identify the QPP-like spatiotemporal mode within each participant.

Consistent with prior work demonstrating that the canonical QPP is characterized by anti-correlated activity between default mode and externally oriented systems like DAN (Bolt et al., 2022), candidate components were screened among the first three complex principal components after exclusion of globally synchronous components. The selected QPP component was defined as the non-global component exhibiting the strongest DMN-versus-DAN opposition, quantified using a combination of relative amplitude separation and phase offset across large-scale networks derived from a BASC122-to-Yeo mapping.

Importantly, this selection criterion was predefined and applied identically across participants, without reference to SAS-M group, behavioral scores, or between-group effects. Thus, selection was hypothesis-informed with respect to the canonical spatial organization of the QPP but independent of subsequent group comparisons. However, because DMN-DAN opposition contributed to identifying the subject-specific QPP-like component and DMN-DAN relationships were subsequently quantified within that component, these measures are not fully independent of the selection criterion. The resulting DMN-DAN analyses therefore test whether the expression of this predefined QPP-like pattern differs between groups, rather than providing an unconstrained test of DMN-DAN organization.

This procedure was intended to identify the dominant subject-specific component most consistent with the canonical DMN-DAN QPP organization described previously using cPCA-based approaches (Bolt et al., 2022). Importantly, the selection procedure did not impose a fixed magnitude of DMN-DAN opposition or network configuration across subjects, but rather identified the non-global component exhibiting the strongest relative DMN-attention-system separation within each individual dataset. Subsequent analyses therefore quantified variability in the spatiotemporal organization, network participation, and state-dependent behavior of the selected QPP-like mode across subjects and groups.

In more detail, QPP-like mode selection focused on the first three complex principal components, which capture the majority of structured variance in resting-state data (Bolt et al., 2022). Global similarity was computed as the absolute cosine similarity between each complex component and a spatially uniform vector. Components were excluded if they were the most globally synchronous component or if global similarity exceeded 0.75. Among the remaining candidate components, the QPP score was computed as DMN-DAN opposition magnitude weighted by normalized DMN-DAN phase separation, and the highest-scoring component was selected. Components with DMN-DAN phase separation < 2.2 radians, opposition magnitude < 0.05, or composite score < 0.05 were classified as weak; components with no eligible candidate were classified as absent. In the present dataset, all subjects exhibited identifiable QPP or weak-QPP components (Supplementary Figure 2).

### 2.10 QPP sign alignment for network analysis

Because complex principal components are defined up to an arbitrary sign, the orientation of QPP components can vary across subjects, leading to apparent inversions of network activity (e.g., DMN up vs. down) that are not meaningful. This is another consideration not encountered when running cPCA on concatenated group-level scans [such as in Bolt et al. (2022)]. To ensure network interpretability across subjects, we sign-aligned QPP components prior to analysis. For subject-level analyses, the selected QPP component was oriented such that the DMN exhibited consistent polarity across participants. This alignment ensured that comparisons of network phase relationships and anticorrelation structure (e.g., DMN vs. attention networks) reflected true differences in spatiotemporal dynamics rather than arbitrary sign differences.

### 2.11 Exploratory subject-wise QPP-attractor visualization

To illustrate the temporal relationship between whole-component QPP expression and attractor-state organization, a subject-specific QPP time course was derived independently for each participant from the selected cPCA component. The real component score was extracted at each TR, sign-aligned to maintain consistent DMN polarity across participants, z-scored, and temporally matched to the corresponding attractor-state labels. Example time courses and group-averaged network trajectories are presented descriptively in Supplementary Figure 4 to illustrate the relative timescales of continuous QPP fluctuations and discrete attractor-state transitions.

### 2.12 Subject-wise QPP network behavior vs. attractor states

To characterize network-resolved QPP dynamics across attractor states (Figure 5), subject-specific QPP time courses were reconstructed from each participant’s selected cPCA component, sign-aligned to maintain consistent DMN polarity, and averaged within five networks of interest using the BASC-122-to-Yeo-7 functional-system assignments: DMN, DAN, FPN, VAN, and VIS. Whole-scan network amplitude was quantified as the mean absolute value of each network-specific reconstructed QPP time course. These measures therefore reflect the magnitude of network participation within the QPP component rather than raw BOLD amplitude. Group differences were assessed using Wilcoxon rank-sum and subject-level permutation tests (10,000 permutations), with Cohen’s *d* (High SAS-M minus Low SAS-M) reported as the effect size. To account for the five network-level comparisons, permutation-derived p-values were corrected across DMN, DAN, FPN, VAN, and VIS using the Benjamini-Hochberg FDR procedure.

To examine context-dependent relationships between the DMN and attention/control systems, subsequent pairwise analyses focused on DMN-DAN, DMN-FPN, and DMN-VAN contrasts. VIS was included in the broader network-amplitude analysis but not in these DMN-centered contrasts, which were specifically intended to characterize relationships between the DMN and attention/control networks. Pairwise contrasts were calculated at each TR as the difference between reconstructed network signals. For state-wise analyses, absolute contrasts were z-scored within subject and averaged within each attractor state, thereby quantifying network separation irrespective of polarity. Group differences were assessed using Wilcoxon rank-sum tests, with permutation tests additionally calculated for descriptive robustness. These state-wise comparisons were exploratory and were not intended as an omnibus test of Group by Attractor State by Network-Pair effects. Benjamini-Hochberg FDR correction was applied to the permutation-derived *p*-values across the four attractor-state comparisons separately within each network pair. Because none survived FDR correction, nominal effects are reported for descriptive purposes only and are not interpreted as evidence of state-specific group differences.

For transition-triggered analyses, signed DMN-centered contrasts were z-scored within subject and averaged from −5 to +5 TR around internal-external context and action-perception transitions. Group differences were assessed at each lag using Wilcoxon rank-sum tests, with Benjamini-Hochberg FDR correction applied across lags separately within each network pair and transition type. Nominal (*p* < 0.05) and FDR-corrected (q < 0.05) effects are distinguished in Figure 5.

To test for group differences across the transition trajectory, we also ran a cluster-based permutation test separately for each network pair and transition type (Supplementary Table 2). Adjacent time points from −5 to +5 TR formed a cluster when the absolute group-comparison t statistic reached 2.024. We summed those statistics to measure each cluster’s strength. We then shuffled participants’ group labels 10,000 times while keeping each participant’s full trajectory intact. A cluster’s *p*-value was the proportion of shuffled datasets whose strongest cluster was at least as strong as the observed cluster, with a standard adjustment to avoid a *p*-value of zero.

Transition-triggered analyses included only participants with at least one qualifying transition of the corresponding type for which the complete −5 to +5 TR window was available. Accordingly, internal-external context transition analyses included 34 participants, whereas action-perception transition analyses included 26 participants; participants without a qualifying transition contributed to the other analyses for which data were available.

### 2.13 Parcellation for atlas/network mapping

For network-level interpretation, BASC-122 parcels were assigned approximate Yeo-7 functional-system labels according to their spatial overlap with the Yeo-7 atlas (Thomas Yeo et al., 2011). This mapping was used only to group and summarize BASC-derived signals at the network level. The underlying regional time series were extracted directly using the BASC-122 parcellation and were not spatially reassigned to Yeo parcels. Because BASC-122 and Yeo-7 were derived independently and have non-identical parcel boundaries, these assignments should be regarded as approximate functional-system annotations rather than exact atlas correspondences. Parcels intersecting multiple Yeo systems or lying near network boundaries may therefore be susceptible to assignment ambiguity, such that a parcel assigned to one Yeo-7 network may contain signal from regions associated with an adjacent network. Importantly, this mapping was applied only after extraction of the BASC-122 regional time series and therefore does not introduce spatial resampling or misalignment into the underlying parcel-level signals themselves. The complete parcel-wise mapping and overlap fractions are provided in Supplementary Table 3, while a visual network mapping is shown in Figures 4, 5.

## 3 Results

### 3.1 Qualitative differences in attractor state space

To visualize large-scale attractor organization, subject trajectories were projected into a two-dimensional space defined by internal-external context and perception-action dimensions (Figure 2A). Both groups exhibited similar state-space coverage, with the greatest density of time points near the origin. Subject-level mean positions are shown at an expanded scale in Figure 2B. Both group centroids remained close to the origin, although the High SAS-M group showed a modest descriptive shift toward the internal-context (left quadrant, Figure 2A) and action directions (upper quadrant) and greater dispersion (Figure 2B). These visual differences were descriptive and were not interpreted as evidence of statistically reliable group separation.

### 3.2 Attractor state dynamics

Subject-level comparisons of attractor-state occupancy did not reveal robust group differences. An omnibus permutation test comparing four-state occupancy vectors between groups was not significant (*p* = 0.454; 10,000 permutations), and no subject-level Wilcoxon rank-sum tests reached nominal significance for state fractions, axis scores, mean dwell times, state entropy, or switch rate (all *p* > 0.05).

The largest non-significant trend was for external-context fraction, which was lower in the High SAS-M group than the Low SAS-M group (means 0.225 vs. 0.276, *p* = 0.121, Cohen’s *d* = −0.48, Cliff’s delta = −0.29). Mean external-context dwell time showed a similar trend (*p* = 0.071, *d* = −0.54), whereas mean action dwell time showed only a modest effect (*p* = 0.308, *d* = 0.39). State entropy and switch rate were closely comparable between groups (*p* = 0.455 and 0.744, respectively), arguing against a strong group difference in overall switching dynamics or attractor-state diversity. Levene’s test suggested unequal variance only for action dwell time (*p* = 0.036), while all other variance comparisons were non-significant.

### 3.3 Attractor state and QPP dynamics vs. SAS-M and behavior scores

Exploratory continuous associations were examined between behavioral questionnaire scores and subject-level attractor states, as well as QPP amplitude measures (Supplementary Figure 1 and Supplementary Table 1). Associations between questionnaire scores and attractor-state metrics yielded several nominally significant relationships, but none survived correction for multiple comparisons (Supplementary Table 1). Similarly, no associations between SAS-M scores and average QPP network amplitudes survived correction (Supplementary Figure 1C).

In contrast, analyses of SAS-M vs. internal-external context transitions by subject showed nominal positive associations between SAS-M scores and DMN-DAN contrast at lags −1 and +1 surrounding External-Internal context transitions. Notably, these are the same lags at which categorical average group differences were observed in Figure 5C. These associations did not survive FDR correction. SAS-M scores were also positively associated with DMN-FPN contrast surrounding External-Internal transitions, with Spearman correlations surviving FDR correction at lags −1, 0, +1, and +3 (Supplementary Figures 1A, B). These behavioral correlations are addressed further in the discussion section and are interpreted cautiously given the modest sample size and exploratory nature of the analyses.

### 3.4 Exploratory regional activity by attractor state

Exploratory state-specific comparisons revealed modest and spatially heterogeneous differences between High and Low SAS-M groups. At the network level, a small number of nominal effects were observed (permutation *p* < 0.05), primarily within the external-context state, including higher dorsal attention network activity and lower somatomotor activity in the High SAS-M group. However, no network-level comparison survived FDR correction within each state. Parcel-wise analyses similarly showed distributed moderate effect sizes across multiple states, particularly the internal-context, action, and external-context states. Several parcels reached nominal significance (*p* < 0.05), but none survived within-state FDR correction.

Accordingly, these state-specific network- and parcel-level findings are considered exploratory and do not provide corrected statistical evidence for group differences in state-dependent regional activity.

### 3.5 Exploratory group-level QPP network analysis

Exploratory group-template reconstructions showed qualitative differences in the reconstructed DMN and attention-network trajectories between the High and Low SAS-M groups (Supplementary Figure 3). Because these trajectories reflect both amplitude and temporal variation, their relative separation cannot be interpreted as a direct measure of phase offset. Moreover, because they were derived from group-level cPCA templates rather than independent subject-level estimates, the figure is presented descriptively and is not interpreted as evidence of an inferential group effect.

### 3.6 QPP network-level expression vs. attractor states and transitions

To characterize network-resolved QPP dynamics, we examined whole-scan network amplitudes, differences between DMN activity and activity in each attention network within attractor states, and those same differences surrounding state transitions (Figure 5). Mean QPP amplitude was lower in the High SAS-M group for DAN (rank-sum *p* = 0.0187; permutation *p* = 0.0325; *d* = −0.68) and VIS (permutation *p* = 0.0370; *d* = −0.69). These measures reflect participation within the reconstructed QPP component, rather than overall BOLD activity. Neither difference survived FDR correction across the five networks.

State-wise analyses yielded nominally greater absolute DMN-DAN separation in the High SAS-M group during the internal-context state (*p* = 0.0328, *d* = 0.52) and less DMN-FPN separation during the external-context state (*p* = 0.0401, *d* = −0.63). Neither effect was supported by permutation testing or survived FDR correction.

The clearest differences occurred around internal-external context transitions. The High SAS-M group showed greater signed DMN-DAN contrast at lags -1 (*p* = 0.0083, q = 0.0457) and +1 (*p* = 0.0018, q = 0.0197). Cluster-based permutation tests also identified DMN-DAN differences spanning −3 to −1 TR (cluster *p* = 0.031) and +1 to +3 TR (*p* = 0.032), as well as a DMN-FPN difference spanning −1 to +5 TR (*p* = 0.005). No significant clusters were found for DMN-VAN or action-perception transitions. Complementary continuous analyses related SAS-M scores to transition-associated DMN contrasts (Supplementary Figure 1): DMN-DAN associations at −1 and +1 TR were nominal, while several DMN-FPN associations survived FDR correction across lags. These continuous associations should be interpreted cautiously given the modest sample size and exploratory nature of the analyses. Evidence for a group difference is strongest around internal-external transitions. The nominal whole-scan DAN/VIS and state-wise findings should not be interpreted as corrected group effects.

## 4 Discussion

The present findings provided partial support for our hypotheses and suggest that differences associated with higher smartphone addiction scores were more apparent in large-scale spatiotemporal network dynamics than in attractor-state occupancy. Contrary to our first hypothesis, High SAS-M participants did not show broadly reduced DMN-DAN or DMN-FPN anticorrelation in subject-level QPP-like dynamics. However, our second hypothesis received stronger support. Differences in DMN-external-attention-network relationships emerged specifically surrounding internal-external attractor-state transitions, including FDR-corrected DMN-DAN differences and additional DMN-FPN effects supported by trajectory-level and continuous analyses but less consistently by lag-wise group comparisons. Across analyses, the clearest effects therefore emerged in the network-resolved QPP results, particularly in altered participation of attention-related systems and increased DMN dominance around internal-external context transitions. In contrast, attractor-state occupancy and subject-level summary metrics were broadly similar between groups.

### 4.1 Attractor state summary

Analysis of attractor-state dynamics revealed no robust group differences in overall state occupancy or subject-level summary metrics. Nevertheless, qualitative examination of the attractor-state space suggested a tendency for individuals with higher SAS-M scores to occupy regions associated with action-oriented states and to exhibit broader dispersion within the attractor landscape. The state-specific activities by region (Figure 4) revealed trends for elevated engagement in regions such as occipital visual areas during external states and bilateral amygdala in internal states (Figure 4C), but these trends did not survive multiple comparisons correction.

### 4.2 Group-level QPP summary

At the group level, separately reconstructed QPP templates showed qualitative differences in DMN, DAN, and FPN trajectories between the High and Low SAS-M groups (Supplementary Figure 3). These trajectories reflect both amplitude and timing, so their visual separation cannot establish a difference in relative phase. Because the templates do not provide independent subject-level estimates, we present this comparison descriptively and draw no group-level inference from it.

### 4.3 Subject-specific-QPP component vs. attractor states

Whole-component QPP expression did not show reliable group differences across attractor states. This provides limited support for a generalized alteration of QPP-attractor coupling and instead suggests that group effects, where present, are more specific to network-resolved or transition-dependent aspects of QPP dynamics.

### 4.4 QPP network expression by attractor state

The clearest group differences occurred around internal-external attractor transitions. High SAS-M participants showed greater DMN dominance relative to the DAN immediately before and after these transitions; the lag-wise effects survived FDR correction. DMN-FPN differences were less consistent at individual lags, although the trajectory-level cluster test identified a significant effect. We found no comparable cluster for DMN-VAN or action-perception transitions.

Whole-scan DAN and VIS participation in the reconstructed QPP was lower in the High SAS-M group at nominal thresholds, but neither difference survived FDR correction. State-wise DMN-DAN and DMN-FPN contrasts were likewise exploratory and did not survive correction. Together, the results point most strongly to differences surrounding internal-external transitions, rather than broad differences in attractor-state occupancy or QPP network amplitude.

### 4.5 Synthesis of results

Together, the present findings suggest that high smartphone addiction scores (SAS-M) are associated more strongly with altered attention-network dynamics during cognitive-state transitions than with large differences in general resting-state dynamics. The clearest effects emerged during transitions between internal and external attractor states, where individuals with higher SAS-M scores showed increased DMN dominance relative to attention-related systems, particularly the DAN, during subject-specific QPP dynamics.

Several of these features, particularly increased DMN-attention-network separation, resemble features of attention previously reported during task-engaged or externally directed cognitive states in QPP studies (Seeburger et al., 2024; Watters et al., 2025). Recent work combining QPP analysis with task fMRI further demonstrated that decoupling between the DMN and FPN is associated with elevated cognitive demand during working-memory tasks (Seeburger et al., 2026). In the present study, qualitatively similar patterns were observed during resting-state activity in High SAS-M subjects, including DMN-FPN separation and altered DMN-attention-network relationships. These effects were modest and not uniformly robust across analyses.

Within the broader framework of attentional control theory (Eysenck and Derakshan, 2011), efficient attentional shifting depends on the limited resources of executive control networks, including the FPN and DAN, which support top-down attentional regulation and goal-directed processing through interactions with the DMN (Dixon et al., 2018). Although attentional control theory was originally developed in the context of anxiety-related attentional bias, the broader framework may also be relevant to persistent high-stimulation digital engagement, where frequent switching between salient stimuli could place sustained demands on executive control systems. In this context, increased DMN dominance relative to the DAN and FPN surrounding internal-external attractor transitions may reflect reduced efficiency in reallocating attentional resources during shifts in cognitive context. However, this interpretation remains speculative and cannot be directly inferred from resting-state data alone. Future work combining task-based paradigms, objective smartphone-use measures, and dynamic network analyses will be necessary to determine whether these transition-associated QPP differences correspond to measurable alterations in attentional control or executive flexibility.

The differing results obtained with fcANN and cPCA/QPP analyses likely reflect the distinct features of brain dynamics captured by the two approaches. The fcANN framework uses each participant’s functional connectivity to model a set of large-scale brain activity states and then characterizes how the BOLD signal moves among and occupies those states over time, whereas cPCA directly identifies recurring spatiotemporal patterns in the empirical BOLD signal and permits examination of network-specific QPP expression. Thus, relatively preserved attractor-state organization between groups can coexist with alterations in the amplitude or temporal expression of network dynamics within and surrounding those states. In the present data, the stronger QPP findings suggest that SAS-M-related differences may be more apparent in transient, network-resolved dynamics than in coarse-grained attractor-state occupancy or switching measures. In particular, the altered DMN-DAN and DMN-FPN QPP contrasts surrounding internal-external attractor transitions may indicate differences in the coordination of internally and externally oriented attentional systems during periods of cognitive-state reorganization.

Given the modest sample size and exploratory nature of several analyses, these findings should be interpreted as hypothesis-generating. Nevertheless, they provide a preliminary framework for understanding how habitual engagement with high-stimulation digital environments may relate to alterations in intrinsic large-scale brain dynamics. Future work with larger samples and longer acquisitions will be necessary to determine whether these effects represent stable trait-level differences or context-dependent adaptations to patterns of digital engagement.

### 4.6 Limitations

Several limitations should be considered when interpreting the present findings. First, head motion was addressed through exclusion of high-motion participants in the original dataset (Abdul Rashid et al., 2021) and regression of motion parameters during preprocessing in the CONN pipeline. Remaining subjects did not differ significantly in framewise displacement between groups. Nevertheless, residual motion effects cannot be fully excluded and may subtly influence dynamic fMRI measures.

Second, the sample size was modest (*N* = 40), limiting statistical power and likely contributing to the observation that several moderate to strong effects did not survive correction for multiple comparisons. Neuroimaging measures, especially functional correlation, are notoriously susceptible to poor replicability and generalizability in small sample sizes (Marek and Laumann, 2025). While we applied dynamic measures such as fcANN that show robustness across datasets (Englert et al., 2025), these findings are susceptible to the same fMRI pitfalls and should be interpreted as exploratory and hypothesis-generating rather than definitive evidence of group differences.

Third, QPP detection at subject-level was performed on relatively short resting-state scans, which may reduce the stability and reliability of estimated spatiotemporal patterns. This highlights the need for longer acquisitions or replication in larger datasets. More broadly, while subject-specific identification of QPP-like components may provide a useful framework for studying inter-individual variability in large-scale spatiotemporal dynamics, the reliability and optimal selection criteria for individual-level cPCA-derived QPP estimation remain areas for further methodological validation.

Fourth, while attractor-based and QPP approaches provide insights into dynamic brain organization, both rely on modeling assumptions (e.g., predefined attractor embeddings, component selection criteria in cPCA) that may influence results. Future work using alternative dynamic analysis frameworks would help establish the robustness and generalizability of the observed effects. In addition, the cross-sectional design precludes causal inference regarding the directionality of the relationship between smartphone use and brain dynamics. Also, although identical component-selection criteria were applied across participants independently of group membership, interindividual variability in cPCA component ordering and expression could introduce additional variance into subject-level QPP estimates.

Smartphone addiction was assessed using self-report questionnaire measures, which may be subject to reporting bias and may not fully capture actual usage behavior (Coyne et al., 2023). Objective measures of smartphone use (e.g., screen time logs, app usage metrics) would provide a more precise characterization of digital engagement and its relationship to brain dynamics. The categorical grouping of participants according to SAS-M scores is not ideal. High and Low SAS-M classifications were inherited from the original study and were consistent with the previously validated SAS-M screening threshold (>98; Ching et al., 2015), rather than being derived post hoc from the present imaging data. Nevertheless, SAS-M scores represent a continuous self-report measure, and dichotomization necessarily reduces information about variation within groups, particularly for participants near the screening threshold. We therefore additionally examined continuous associations between SAS-M scores and subject-level imaging measures (Supplementary Figure 1 and Supplementary Table 1). Continuous analyses provided some convergent evidence for the internal-external transition associated QPP findings: subject-level SAS-M scores were nominally associated with DMN-DAN contrast at the same −1 and +1 lags surrounding External-Internal context transitions at which categorical group differences were observed, although these associations did not survive FDR correction. However, associations between SAS-M scores and DMN-FPN contrast were also observed around External-Internal transitions, with several Spearman correlations surviving FDR correction across lags. These findings suggest that some transition-associated effects are not solely dependent on categorical classification; however, given the modest sample size, multiple comparisons, and reliance on a self-report screening measure, the continuous associations should also be interpreted cautiously. The present findings therefore should not be interpreted as establishing a discrete neurobiological boundary between High and Low SAS-M individuals. Future studies using larger samples together with continuous and objective measures of smartphone use will be important for establishing the robustness and functional significance of these relationships.

Transition-triggered analyses also involved reduced sample sizes because some participants did not exhibit attractor state transitions during the scan; notably, all six participants without a usable internal-external context transition were in the Low SAS-M group. Transition-related findings should therefore be interpreted with this sampling characteristic in mind.

Finally, as with the original study of this dataset, no significant group differences in age or sex distribution were identified, although the sample size limits excluding demographics as a confound. Future studies with larger samples and objective behavioral measures will be important for evaluating the specificity of the reported effects (see below).

### 4.7 Network mappings

Network-level findings should also be interpreted in light of the approximate mapping between BASC-122 parcels and Yeo-7 functional systems. Because the two atlases were independently derived and have nonidentical boundaries, BASC parcels spanning multiple Yeo systems may be imperfectly represented by a single network assignment, potentially introducing noise or regional misclassification into network-level summaries. More generally, atlas-based assignment of functional networks necessarily assumes correspondence between group-level network boundaries and their expression in individual participants, despite known interindividual variability in functional network topography (Gordon et al., 2017). The BASC-to-Yeo mapping therefore represents an additional source of assignment uncertainty beyond this inherent limitation of group-level parcellation. Importantly, this mapping does not affect the underlying BASC-122 time-series extraction, but may influence the network-level attribution of effects. Accordingly, parcel-wise assignments and overlap fractions are provided in Supplementary Table 3, and replication using alternative or individually defined network parcellations would help establish the robustness of the network-specific findings.

### 4.8 QPP component ranks and DMN-DAN opposition strength

Although the High SAS-M group showed a descriptive tendency toward weaker and higher-rank QPP components (Supplementary Figure 2), the distribution of selected component ranks did not significantly differ between groups (χ^2^ *p* = 0.237). Variability in component rank and QPP strength may reflect a combination of true interindividual differences in the prominence of QPP dynamics and variability in subject-level cPCA decomposition. Thus, although the absence of a significant group difference in component rank provides no evidence for a systematic difference in decomposition across groups, we cannot fully exclude the possibility that decomposition quality or signal-to-noise differences contribute to variability in the observed network-amplitude effects. Identical component-selection criteria were applied across participants, but replication in larger samples will be important for determining the reliability of subject-level QPP decomposition and network-amplitude estimates. Because DMN-DAN opposition contributed to component selection, subsequent DMN-DAN measures are partly dependent on this criterion, representing an additional limitation of the subject-specific approach.

Future validation using independently defined QPP templates, cross-validated component estimation, or multicomponent representations would help determine the extent to which the present findings generalize beyond the subject-specific component-selection procedure used here.

### 4.9 Smartphone use as a consideration in neuroimaging studies

Objective measures of smartphone use are rarely collected or reported in neuroimaging datasets of smartphone addiction, often relying on self-report/questionnaire data instead (Korte, 2020). The present findings, together with prior work, suggest that excessive smartphone use may contribute to unmeasured heterogeneity in fMRI studies more broadly, beyond studies specifically focused on behavioral addiction. Given the growing body of behavioral and neuroimaging evidence linking problematic smartphone use to alterations in attention, cognitive control, and cortical network organization (Liebherr et al., 2020; Anbumalar and Binu Sahayam, 2024), it may be important to consider smartphone usage as a standard demographic variable in future neuroimaging studies.

Large scale consortia neuroimaging efforts such as the Human Connectome Project and UK Biobank already document demographic and behavioral data on age, sex, intelligence, sleep, substance use, psychiatric history, etc., (Glasser et al., 2016; Miller et al., 2016). Incorporating objective measures such as screen time, social media time, or interaction frequency into large-scale consortia efforts could help clarify the brain-behavior effects of smartphone addiction. This is especially important given the aforementioned precarity of small sample neuroimaging studies, which may require sample sizes into the hundreds before small effects and correlations are replicable and generalizable (Marek and Laumann, 2025). Given the need for longer scan times and higher sample sizes (Ooi et al., 2025), small-scale studies focused exclusively on smartphone addiction may face substantial challenges in achieving the sample sizes needed for robust and generalizable brain-behavior inference.

## 5 Conclusion and future directions

In summary, the clearest effects observed in the present study involved altered coordination between default mode and attention networks during transitions between internally and externally oriented attractor states. Individuals with higher SAS-M scores showed increased DMN dominance and reduced participation of attention-related networks, particularly during subject-specific QPP dynamics. Although several findings were exploratory or modest in isolation, a pattern across the complementary dynamic fMRI analyses suggested altered state-dependent organization of large-scale resting-state brain dynamics related to high SAS-M scores. This is consistent with evidence that brain network dynamics may serve as transdiagnostic and dimensional biomarkers of psychiatric and psychological disorder (Cocuzza et al., 2026).

These findings are preliminary and hypothesis-generating, but support further investigation of the relationship between excessive smartphone use and resting-state brain dynamics and the coordination of attention networks. Future work incorporating longitudinal designs, objective smartphone usage metrics, and task-based paradigms will be critical for establishing causality and clarifying the functional significance of these effects.

As digital technologies continue to shape daily cognitive experience, understanding their relationship to intrinsic brain dynamics will be increasingly important for both basic neuroscience and mental health research.

## Data Availability

Attractor network dynamic analysis used the publicly available code from Englert et al. (2025), (repository: https://github.com/pni-lab/connattractor). Complex principal component analysis was performed using the publicly available implementation released by Bolt et al. (2022) (repository: https://github.com/tsb46/complex_pca) for fMRI analyses. The open-source SPM-based functional connectivity toolbox (CONN) was used in MATLAB for all preprocessing (Whitfield-Gabrieli and Nieto-Castanon, 2012). Nilearn tools were used for interaction with the Connattractor toolbox and some data visualization (https://nilearn.github.io). All additional code used for preprocessing, analyses, and figure generation was developed in-house using Python 3.14 (Python Software Foundation) and MATLAB R2024a (MathWorks, Natick, MA, United States). The main analysis code is available at: https://github.com/Dalimear/smartphone-qpp-cPCA-attractor-dynamics. The raw or preprocessed fMRI scans may be made available upon reasonable request from the original study authors.
