# Supplementary material for: Altered attention network dynamics in resting-state fMRI of individuals with high smartphone addiction scores
Source: Front Neurosci. 2026 Oct 14;20:1948718. doi: 10.3389/fnins.2026.1948718 (PMC13713798; doi:10.3389/fnins.2026.1948718)
Supplement: Supplementary file 1 [file Supplementary_file_1.docx]

Supplementary Figures and Table

**Supplementary Figure 1.** Continuous associations between SAS-M and behavioral scores and QPP-derived measures. **A)** Representative associations between continuous SAS-M scores and DMN-DAN and DMN–FPN QPP contrasts surrounding External–Internal context transitions. **B)** All External-Internal context-transition associations reaching nominal significance (p < .05) for either Pearson or Spearman correlation; ** indicates q < .05 following FDR correction across lags within each network contrast. **C)** Correlations between behavioral scores and whole-scan QPP network amplitudes.

**Supplementary Figure 2**: A) Fraction of strong vs weak QPPs. Note, all subjects had at least weak QPPs. B) Distribution of selected QPP components by cPCA rank. Although the High SAS-M group showed a descriptive shift toward selection of higher-rank components, rank distributions did not significantly differ between groups (χ² p=.237, V=.27). Component rank may reflect both methodological variability in subject-level decomposition and interindividual variation in the relative prominence of QPP dynamics.

**Supplementary Figure 3**: Exploratory group-level QPP network trajectories. Network trajectories were reconstructed from separate group-level cPCA templates and aligned to the DMN within each group. Qualitative differences in reconstructed network trajectories are visible between the High and Low SAS-M templates; however, because amplitude and temporal variation are not independently quantified in this visualization, relative vertical separation should not be interpreted as a direct measure of phase offset. Because these trajectories are derived from group-level templates rather than independent subject-level estimates, the figure is presented descriptively and no inferential statistics are reported.

**Supplementary Figure 4:** Descriptive visualization of subject-level QPP expression relative to attractor-state dynamics. Subject-specific QPP expression was derived from each participant’s selected cPCA component, sign-aligned across participants, and temporally matched to attractor-state labels at each TR. The figure is intended to illustrate the relative timescales of discrete attractor-state transitions and continuous infraslow QPP fluctuations; subject-level analyses did not identify significant between-group differences in whole-component QPP expression across attractor states. **A)** Example participant time courses showing z-scored QPP component expression overlaid with attractor-state labels. Colored background segments indicate internal context (blue), action (red), external context (yellow), and perception (green), illustrating that attractor-state assignments can change multiple times over the course of an infraslow QPP fluctuation. **B)** Group-averaged QPP network trajectories across one phase cycle (0–2π), shown separately for High and Low SAS-M groups. Lines represent mean signed contributions from the default mode (DMN), dorsal attention (DAN), and frontoparietal (FPN) networks, with shading indicating ±SEM across participants. Background colors indicate the dominant attractor-state assignment at each QPP phase. Panel B is provided as a descriptive visualization of the temporal relationship between network-level QPP expression and attractor-state organization; no significant between-group differences in whole-component QPP-state coupling were identified.

**Supplementary Table 1**: Summary of Attractor State Metrics vs Subject Behavioral Scores. Note that while some trends were observed with respect to smartphone addiction and depression-anxiety scores, no correlations reached corrected significance. No other metrics vs behavioral score correlations survived correction for multiple comparisons.

**Supplementary Table 2**: Cluster-based permutation analysis of transition-triggered QPP trajectories. To account for temporal dependence among adjacent lags, group differences in network-pair trajectories from −5 to +5 TR surrounding attractor-state transitions were evaluated using cluster-based permutation testing. Contiguous lags exceeding a two-sided cluster-forming threshold (|t| ≥ 2.024) were grouped into temporal clusters, with cluster mass calculated as the summed absolute t-statistic across constituent lags. Statistical significance was determined using 10,000 subject-level permutations of group labels, preserving each participant’s complete temporal trajectory, with inference based on the maximum cluster mass within each network-pair × transition-type analysis. Significant clusters were observed surrounding internal–external context transitions for DMN-DAN from −3 to −1 TR (p = .031) and +1 to +3 TR (p = .032), and for DMN-FPN from −1 to +5 TR (p = .005). No significant clusters were observed for action-perception transitions or DMN-VAN. Network-pair/transition combinations without supra-threshold clusters are not displayed.

| BASC-122 parcel | Yeo-7 index | Yeo-7 network | Overlap fraction |
| --- | --- | --- | --- |
| 1 | 2 | Somatomotor | 0.757 |
| 2 | 5 | Limbic | 0.007 |
| 3 | 1 | Visual | 0.200 |
| 4 | 7 | Default | 0.821 |
| 5 | 2 | Somatomotor | 0.609 |
| 6 | 4 | VentralAttention | 0.527 |
| 7 | 6 | Frontoparietal | 0.371 |
| 8 | 2 | Somatomotor | 0.833 |
| 9 | 0 | Unassigned | NaN |
| 10 | 0 | Unassigned | NaN |
| 11 | 0 | Unassigned | NaN |
| 12 | 4 | VentralAttention | 0.555 |
| 13 | 6 | Frontoparietal | 0.002 |
| 14 | 0 | Unassigned | NaN |
| 15 | 7 | Default | 0.573 |
| 16 | 1 | Visual | 0.641 |
| 17 | 7 | Default | 0.825 |
| 18 | 0 | Unassigned | NaN |
| 19 | 1 | Visual | 0.644 |
| 20 | 3 | DorsalAttention | 0.483 |
| 21 | 4 | VentralAttention | 0.495 |
| 22 | 3 | DorsalAttention | 0.409 |
| 23 | 4 | VentralAttention | 0.839 |
| 24 | 7 | Default | 0.675 |
| 25 | 4 | VentralAttention | 0.019 |
| 26 | 4 | VentralAttention | 0.527 |
| 27 | 6 | Frontoparietal | 0.339 |
| 28 | 2 | Somatomotor | 0.406 |
| 29 | 1 | Visual | 0.879 |
| 30 | 7 | Default | 0.448 |
| 31 | 0 | Unassigned | NaN |
| 32 | 7 | Default | 0.792 |
| 33 | 7 | Default | 0.492 |
| 34 | 6 | Frontoparietal | 0.465 |
| 35 | 7 | Default | 0.673 |
| 36 | 0 | Unassigned | NaN |
| 37 | 3 | DorsalAttention | 0.765 |
| 38 | 4 | VentralAttention | 0.394 |
| 39 | 1 | Visual | 0.667 |
| 40 | 1 | Visual | 0.813 |
| 41 | 1 | Visual | 0.773 |
| 42 | 1 | Visual | 0.026 |
| 43 | 1 | Visual | 0.718 |
| 44 | 1 | Visual | 0.021 |
| 45 | 0 | Unassigned | NaN |
| 46 | 0 | Unassigned | NaN |
| 47 | 3 | DorsalAttention | 0.381 |
| 48 | 1 | Visual | 0.004 |
| 49 | 3 | DorsalAttention | 0.554 |
| 50 | 3 | DorsalAttention | 0.306 |
| 51 | 1 | Visual | 0.754 |
| 52 | 2 | Somatomotor | 0.527 |
| 53 | 7 | Default | 0.625 |
| 54 | 0 | Unassigned | NaN |
| 55 | 7 | Default | 0.792 |
| 56 | 1 | Visual | 0.729 |
| 57 | 1 | Visual | 0.819 |
| 58 | 6 | Frontoparietal | 0.365 |
| 59 | 6 | Frontoparietal | 0.443 |
| 60 | 1 | Visual | 0.779 |
| 61 | 5 | Limbic | 0.795 |
| 62 | 0 | Unassigned | NaN |
| 63 | 7 | Default | 0.778 |
| 64 | 3 | DorsalAttention | 0.630 |
| 65 | 0 | Unassigned | NaN |
| 66 | 7 | Default | 0.459 |
| 67 | 2 | Somatomotor | 0.642 |
| 68 | 2 | Somatomotor | 0.716 |
| 69 | 3 | DorsalAttention | 0.500 |
| 70 | 5 | Limbic | 0.698 |
| 71 | 5 | Limbic | 0.118 |
| 72 | 1 | Visual | 0.367 |
| 73 | 2 | Somatomotor | 0.731 |
| 74 | 6 | Frontoparietal | 0.485 |
| 75 | 5 | Limbic | 0.370 |
| 76 | 3 | DorsalAttention | 0.600 |
| 77 | 7 | Default | 0.470 |
| 78 | 5 | Limbic | 0.207 |
| 79 | 1 | Visual | 0.648 |
| 80 | 6 | Frontoparietal | 0.819 |
| 81 | 1 | Visual | 0.804 |
| 82 | 7 | Default | 0.831 |
| 83 | 3 | DorsalAttention | 0.700 |
| 84 | 1 | Visual | 0.133 |
| 85 | 4 | VentralAttention | 0.479 |
| 86 | 5 | Limbic | 0.472 |
| 87 | 7 | Default | 0.747 |
| 88 | 2 | Somatomotor | 0.682 |
| 89 | 7 | Default | 0.778 |
| 90 | 0 | Unassigned | NaN |
| 91 | 7 | Default | 0.321 |
| 92 | 6 | Frontoparietal | 0.750 |
| 93 | 2 | Somatomotor | 0.552 |
| 94 | 6 | Frontoparietal | 0.635 |
| 95 | 4 | VentralAttention | 0.513 |
| 96 | 3 | DorsalAttention | 0.256 |
| 97 | 1 | Visual | 0.002 |
| 98 | 7 | Default | 0.448 |
| 99 | 2 | Somatomotor | 0.741 |
| 100 | 3 | DorsalAttention | 0.334 |
| 101 | 1 | Visual | 0.769 |
| 102 | 3 | DorsalAttention | 0.441 |
| 103 | 7 | Default | 0.483 |
| 104 | 3 | DorsalAttention | 0.465 |
| 105 | 6 | Frontoparietal | 0.525 |
| 106 | 7 | Default | 0.516 |
| 107 | 1 | Visual | 0.724 |
| 108 | 0 | Unassigned | NaN |
| 109 | 5 | Limbic | 0.288 |
| 110 | 2 | Somatomotor | 0.667 |
| 111 | 6 | Frontoparietal | 0.747 |
| 112 | 5 | Limbic | 0.552 |
| 113 | 3 | DorsalAttention | 0.458 |
| 114 | 7 | Default | 0.459 |
| 115 | 7 | Default | 0.698 |
| 116 | 2 | Somatomotor | 0.682 |
| 117 | 3 | DorsalAttention | 0.331 |
| 118 | 4 | VentralAttention | 0.384 |
| 119 | 2 | Somatomotor | 0.471 |
| 120 | 7 | Default | 0.531 |
| 121 | 0 | Unassigned | NaN |
| 122 | 4 | VentralAttention | 0.418 |

**Supplementary Table 3**: Approximate BASC-122 parcel assignments to Yeo-7 functional networks. The overlap fraction indicates the proportion of each BASC parcel assigned to the listed network; assignments were used for network-level summaries and did not alter the extracted parcel time series.
